# Using video reflexive ethnography to explore the use of variable rate intravenous insulin infusions

**DOI:** 10.1186/s12913-022-07883-w

**Published:** 2022-04-23

**Authors:** Mais Iflaifel, Rosemary Lim, Clare Crowley, Francesca Greco, Rick Iedema

**Affiliations:** 1 Reading School of Pharmacy, University of Reading, Reading, Berkshire, UK; 2 Centre for Team Based Practice & Learning in Health Care, King’s College London, London, UK

**Keywords:** Hyperglycaemia, Variable rate intravenous insulin infusions, Video reflexive ethnography, Work as done, Resilient health care, Safety

## Abstract

**Background:**

The use of variable rate intravenous insulin infusion (VRIII) is a complex process that has consistently been implicated in reports of error and consequent harm. Investment in patient safety has focused mainly on learning from errors, though this has yet to be proved to reduce error rates. The Resilient Health Care approach advocates learning from everyday practices. Video reflexive ethnography (VRE) is an innovative methodology used to capture everyday practices, reflect on and thereby improve these. This study set out to explore the use of VRIIIs by utilising the VRE methodology.

**Methods:**

This study was conducted in a Vascular Surgery Unit. VRE methodology was used to collect qualitative data that involved videoing healthcare practitioners caring for patients treated with VRIII and discussing the resulting clips with participants in reflexive meetings. Transcripts of these were subjected to thematic analysis. Quantitative data (e.g. blood glucose measurements) were collected from electronic patient records in order to contextualise the outcomes of the video-observed tasks.

**Results:**

The use of VRE in conjunction with quantitative data revealed that context-dependent adaptations (seeking verbal orders to treat hypoglycaemia) and standardised practices (using VRIII guidelines) were strategies used in everyday work. Reflexive meetings highlighted the challenges faced while using VRIII, which were mainly related to lack of clinical knowledge, e.g. prescribing/continuing long-acting insulin analogues alongside the VRIII, and problems with organisational infrastructure, i.e. the wireless blood glucose meter results sometimes not updating on the electronic system. Reflexive meetings also enabled participants to share the meanings of the reality surrounding them and encouraged them to suggest solutions tailored to their work, for example face-to-face, VRIII-focused training.

**Conclusions:**

VRE deepened understanding of VRIII by shedding light on its essential tasks and the challenges and adaptations entailed by its use. Future research might focus on collecting data across various units and hospitals to develop a full picture of the use of VRIIIs.

**Supplementary Information:**

The online version contains supplementary material available at 10.1186/s12913-022-07883-w.

## Background

Diabetes mellitus is a common, serious, and chronic disorder that affects more than 422 million adults globally [1] and is characterised by hyperglycaemia where fasting blood glucose (BG) levels are greater than 7.0 mmol/L (126 mg/dl) [2]. In the UK, the National Diabetes Inpatient Audit (NaDIA) found that the prevalence of hospitalised adult patients with diabetes has steadily risen from 15% in 2011 to 18% in 2017 [3]. Hyperglycaemia is associated with an increased risk of complications and mortality, a longer hospital stay, and a higher admission rate to the intensive care unit [1, 4].

Variable rate intravenous insulin infusions (VRIII) are considered the treatment of choice to achieve optimal BG levels in patients who are unable to eat, who will miss more than one meal, who have severe illness, e.g. sepsis, and where there are special circumstances, e.g. acute coronary syndrome [2]. VRIII is extremely effective at reducing BG levels quickly for hospitalised patients because of the rapid onset of action of the intravenous route of administration, compared to other administration routes. This characteristic, however, carries the risk of causing patient harm because it is not possible to reverse its action if used inappropriately [5]. Therefore, patients treated with VRIIIs need special care in areas such as regular BG measurements, an adjustment of the VRIII rate according to BG readings, and awareness of the appropriate time to transition from using VRIII to subcutaneous insulin if required. The mismanagement of any of these areas is dangerous and can lead to complications such as hypoglycaemia (BG < 4 mmol/L) and ketoacidosis [3].

Patient safety is a vital concern in hospitals and numerous initiatives have been introduced to enhance patient safety when using VRIIIs. Such initiatives included producing practical guidelines for the use of VRIIIs [6], using prefilled, ready-to-administer, injectable medication to mitigate against patient harm due to preparation errors [7], and recommending independent verification for dose calculations, prescription, dispensing, and administration of certain high-alert medications (e.g. VRIII) in order to identify potential errors before medications are administered to patients [8]. The current initiatives are predominantly based on traditional safety approaches that focus on top-down solutions (e.g. standardised policies) introduced into healthcare systems and/or by external experts by learning from specific errors and producing solutions by eliminating errors and filling in any knowledge gaps (Safety-I) [9]. Healthcare systems are considered complex adaptive systems. By definition, complex adaptive systems are systems that include individual agents − doctors, nurses, pharmacists, and patients − who act and interact with each other and with the surrounding environment (e.g. equipment and technology) in dynamic and unpredictable ways that change over time through learning, feedback, and adaptation [10]. Although great efforts have already been made to enhance patient safety while using VRIIIs, the dependence on traditional safety approaches has proved problematic, failing to demonstrate convincing reductions in risk, error or death [11]. For example, a recent study found that despite the use of strategies to maximise venous access, intensive care unit nurses sometimes ran out of sufficient venous access for VRIIIs. Although the use of workarounds such as prioritising infusions and swapping line of infusion was perceived as important to accomplish the task, the study claimed that these workarounds could decrease patient safety by creating new pathways to VRIIIs administration errors [12].

A different way of thinking about safety was introduced, switching the focus from approaches that concentrate on identifying and eliminating errors to more comprehensive approaches that advocate learning from how things go right as well as how they might go wrong (Safety-II) [13]. A relatively new theory called Resilient Health Care (RHC) adopts the Safety-II way of thinking, focusing on understanding how everyday work is actually done rather than how it is assumed to be done [14]. RHC’s proponents argue that performance variability is an integral part of how people and systems deal with expected and unexpected situations, and that their capacity to successfully adapt is what makes a successful system work, enabling good outcomes in spite of problems and challenges [15]. It suggests a shift in focus from learning only from errors to a more complementary perspective of learning from how things go right as well as how things go wrong [16]. One of the important constructs in RHC is the distinction between how everyday work is actually done (‘Work as Done’, WAD) and how work is *assumed* to be done (‘Work as Imagined’, WAI) [17].

In order to understand how healthcare practitioners work in real life situations (WAD), Iedema et al. used the term ‘exnovation’ (‘innovation from within’) to focus our attention on how healthcare practitioners enact and understand the internal complexity of their clinical work. In contrast to innovation introduced from elsewhere, exnovation is the process of strengthening insight into in situ everyday work, and enabling practitioners to enact on that insight [18]. It is clear that the complexity of care is often taken-for-granted. For that reason, understanding WAD requires an in-depth exploration of everyday work and of the hidden, taken-for-granted clinical work performed by frontline practitioners in situ*.* Video reflexive ethnography (VRE) is a qualitative methodology involving collaboration between researcher and participants and aims at rendering the complexity of ordinary everyday tasks visible through the videoing of everyday work. VRE has three phases: (a) familiarisation with the work through informal observations; (b) video-recording of everyday work; (c) reflexive meetings in which participants and researcher review the edited footage; and (d) implementing insights and ideas produced during reflexive meetings in practice [18]. Video feedback with those involved in the everyday work enables activation of insights articulated by the practitioners, patients, and researchers collaborating to learn about the work, and to make sense of context (reflexivity). Research shows this process results in participants recommending and initiating designing realistic and realisable solutions to enhance safety and care [18].

The present study is part of a project that has a published protocol [19] with the overarching aim of exploring RHC in the use of VRIII. As far as we know, no previous study has used VRE methodology to explore WAD in the use of VRIII. Therefore, the aim of this study was to understand, by using VRE, how VRIIIs are used in a Vascular Surgery Unit.

## Methods

### Study design

The current study was informed by the RHC approach [20, 21] and the four theoretical principles of the VRE methodology: exnovation, reflexivity, collaboration, and care [18]. RHC and VRE principles shaped our ontological stance, which suggested a need for an in-depth understanding of the taken-for-granted clinical tasks associated with the use of VRIIIs. This stance led us to think about the types of knowledge that would be of most value in exploring WAD. We reasoned that it would be important to focus on how clinical work was delivered, and on how adaptations were made in expected and unexpected situations. Therefore, our approach depended mainly on collecting qualitative data using VRE methodology and then reviewing the electronic patient record (EPR) for quantitative data (e.g. BG measurements) to provide context for the qualitative data and clarify the clinical status of the included patients.

### Study setting

The study was conducted in a Vascular Surgery Unit in a tertiary hospital in England that provided emergency and elective treatment for patients with vascular diseases, e.g. peripheral vascular disease and diabetic foot disease. The Unit had 10 patient bays (24 beds), mostly occupied for the duration of the observations. There was a treatment room where the nurses prepared medications and assembled equipment. The Unit used a syringe pump for insulin infusion and a volumetric pump for IV glucose containing fluids. Insulin and glucose containing infusions were administered via a single cannula via a Y-connector with dual anti-reflux valves. The Unit used an electronic prescribing, monitoring, and administration (ePMA) system within the EPR. Consultants, specialist registrars, foundation year one doctors and a nurse in charge met each morning to check patients’ status, examine them and review their progress, laboratory results and medications. Foundation year one doctors discussed with their colleagues on the overnight shift, patients’ developments and documented what consultants and registrars decided as treatment plans during the morning meeting. If foundation year one doctors were busy, the registrar prescribed VRIIIs using the ePMA system. Two nurses conducted independent verification of prescription, patient, infusion pump programming, capillary blood glucose (CBG), VRIII initial rate and for each rate change. There was generally one nurse per six patients and foundation year 1/2 doctors were regularly present.

### Recruitment

Participant recruitment was conducted over 5 months. Based on the published study protocol [19], the aim was to include two patients and all healthcare practitioners responsible for the use of VRIII in their care. Table 1 shows the eligibility criteria and the recruitment process for both patients and healthcare practitioners.

**Table 1:** Eligibility criteria and recruitment process

**Eligibility criteria**	
*Inclusion criteria*	
Healthcare practitioners who are:	
1. Willing to be observed by video recording.	
2. Working in the Vascular Surgery Unit.	
3. Managing/dealing with patients on VRIII.	
Patients who are:	
1. Aged ≥18 years old.	
2. Receiving VRIII for at least 24 h to treat elevated BG.	
3. Under the care of a healthcare practitioners who have consented to participate in this study.	
4. Able to provide informed consent.	
*Exclusion criteria*	
Healthcare practitioners who are:	
1. Not willing to be observed by video recording.	
2. Not working in the Vascular Surgery Unit.	
3. Not involved in the use of VRIII.	
Patients who are:	
1. Not willing to be observed by video recording.	
2. Not prescribed VRIII.	
3. On IV insulin and glucose infusion for hyperkalaemia (potassium levels > 5.5 mmol/L).	
4. Unable to provide informed consent.	
5. Non-English speakers.	
**Recruitment**	
*Healthcare practitioners*	
To recruit potential healthcare practitioners, an invitation letter and participant information sheet outlining the purpose of the study, the methodology, and the design was first sent by the Unit clinical and managerial lead to all potential healthcare practitioners. Then the researcher (MI) met the healthcare practitioners working in the Unit in two ward meetings and explained the study aims and process. Informed consent was then taken from interested healthcare practitioners. A poster with details about the study was also placed in the staff room until the completion of data collection.	
*Patients*	
The study site collaborator with a pharmacist team identified potential patient participants. The researcher confirmed with the senior nurse the appropriateness of the patient before recruiting them. This is in addition to whether the patient had the capacity to consent. Once agreed, the researcher provided an invitation letter and participant information sheet to the patient and explained the purpose and objectives of the study and that as part of filming the work of healthcare practitioners, some parts of their body might appear in the video recordings (arm, leg, etc.). The researcher asked the patient if they had any questions about the study before taking written informed consent.	

### Data collection

#### VRE

The VRE involved three stages:

- Stage 1: Familiarisation and informal observation

There were three aims to this stage: 1) To familiarise the researcher (MI) with the environment and build a level of trust with healthcare practitioners, thus facilitating the video observations. 2) To informally observe the staff working in the Unit, in order to understand how VRIII was being used and in turn determine the tasks that needed to be filmed. 3) To familiarise MI with the ePMA system in order to understand how prescribing, monitoring, documenting and other tasks were being reported when using VRIIIs. This stage involved taking field notes while conducting 15 h of general observations in three to five-hour sessions. It also involved shadowing three healthcare practitioners while they were using VRIII for short periods of 30–60 min each.

- Stage 2: Video observation

The focus of this stage was to capture how VRIII was used in situ. Two digital cameras, one static next to the patient’s bed and the other handheld by the researcher, were used. The static camera focused on the tasks accomplished around the insulin pump. For the tasks that occurred elsewhere, e.g. handover, the assembly of the VRIII and other equipment, preparation and documentation, the handheld camera was used. The video footage was transferred to the project’s shared drive as soon as possible and then deleted from the camera.

- Stage 3: Reflexive meeting discussion

The aim of this stage was to engage the healthcare practitioners collaboratively in analysing their work.

##### Choosing the clips of interest for the reflexive meetings

The research team (MI, RL, CC, FG, RI) and the study site lead nurse (PW) chose the clips for use in the discussion based on the main criteria presented in Table 2. MI is a pharmacist with experience in diabetes management and qualitative research; RL is an experienced academic pharmacist with experience in qualitative methods, system safety and organisational resilience; CC is a hospital pharmacist with considerable experience in safety and quality improvement; RI is a medical sociologist and an early pioneer using VRE in healthcare research studies. FG is an experienced researcher, academic and a pharmacist. The research team embraced reflexivity by acknowledging their common background in diabetes management, VRE use and qualitative research and pooling the personal views that emerged during the research period while compiling notes for reflexive meeting discussions, clips and video observations.

**Table 2:** Criteria used to choose the video clips for use in the reflexive meeting

1. The video clips were chosen on the basis that they could be used to address the research question with the healthcare practitioners, enabling them to describe and scrutinise how VRIII is used in everyday clinical work. The guiding principles of VRE are:	
• Does the footage reveal a range of tasks or a range of different people doing similar things in previously un (der) appreciated ways (reflexivity)?	
• Can the clips show different perspectives on a task and create the possibility for more appropriate understandings, perspectives or solutions (exnovation)?	
• Do the chosen clips and the feedback discussion maintain the psychological safety of the participants (care)?	
• Could the chosen clips help engage healthcare practitioners in collectively identifying safety interventions in the in situ, taken-for-granted everyday work (collaboration)?	
2. The time available for the reflexive session	
• The video clips were chosen in such a way that they could be discussed in 30–45 min.	

##### Conducting the reflexive meetings

In the case of the present study, the hospital site paused all research activity because of the coronavirus (COVID-19) pandemic and the research team responded by agreeing to conduct virtual reflexive meetings with the healthcare practitioners. When it came to organising the reflexive meetings, the aim was to conduct a meeting with all healthcare practitioners who appeared in the video footage. However, it was not feasible to meet all healthcare practitioners, or even a group of two, at the same time. One-to-one reflexive meetings, lasting between 30 and 45 min, were conducted instead. MI conducted reflexive meetings through the Microsoft Teams platform and verbally confirmed continuing consent with healthcare practitioners before the start of the meetings. The strategy for facilitating and running the reflexive meetings was guided by the four principles of VRE [18]. MI was an ‘outsider’ facilitator who used specific clips to facilitate the reflexive meetings in order to achieve specific aims [18]. MI described to the participants the footage they would be watching and facilitated the discussion by asking pre-prepared questions throughout the session (see reflexive meeting discussion guide in see Supplementary file 1) in order to explore what worked well, what might be learned from everyday clinical work, what problems and challenges were faced, and what adaptations and adjustments were made while using VRIIIs. Each of the reflexive meetings was audio-recorded and transcribed verbatim for further analysis.

#### EPR

The EPRs of the patient participants were accessed after completion of videoing to identify CBG measurements, the VRIII regimen selected, the monitoring frequency of CBG and blood ketones, the duration of persistent hyperglycaemia and the number of hypoglycaemic episodes. The quantitative data covered the whole 24 h during which video observations were captured.

### Data analysis

#### VRE

All the video recordings, including verbal utterances and actions, were transcribed and interpreted by MI. Video clips were chosen based on specific criteria (see Table 2) and used in reflexive meetings. Recordings of reflexive meetings were transcribed and quantitative data from EPRs were added to the transcripts to provide a comprehensive written account of the tasks captured in the video recordings, as deemed relevant. For example, if the healthcare practitioner commented on stopping the VRIII because the patient developed hypoglycaemia, the researcher used the quantitative data (BG measurement) to report the exact numerical value of BG that necessitated the withdrawal of VRIII at that time. All the verbatim transcripts of the reflexive meeting discussions were analysed using inductive thematic analysis [22]. MI coded transcripts then constructed sub-themes and themes within and across these codes. In an iterative process, the research team (MI, RL, CC, FG and RI) then met to discuss the overarching themes in order to identify significant broader patterns of meaning that represented how the healthcare practitioners used VRIII.

#### EPR

The quantitative data were collected and compiled by MI and discussed with the study site collaborator (CC) in order to ensure the data collection and reporting processes were robust. The time period during which CBG was at target range was identified. The number of CBG readings of < 4.0 mmol/L (hypoglycaemia), and CBG readings of > 12.0 mmol/L (hyperglycaemia), were recorded. The duration of persistent hyperglycaemia, and the incidence of hypoglycaemia episodes (any CBG reading of < 4.0 mmol/L in a four-hour period), were identified. The number of times the patient required an IV glucose infusion to treat hypoglycaemia was also recorded.

The quantitative data were used to form judgments on the outcomes of the observed tasks and to serve as evidence to support conclusions by adding numerical findings to the transcripts of reflexive meetings.

## Results

Two male patients were recruited and participated in the study. Twenty healthcare practitioners (nurses, senior nurses, pharmacist, specialist registrars, consultants, foundation year one doctors and nurse assistants) working in the Unit provided informed consent before patients were identified and recruited. Twelve healthcare practitioners who were involved in the care of the two patients were approached; of these, 10 healthcare practitioners participated: three nursing assistants, two nurses, three senior nurses, one specialist registrar and one foundation year one doctor. Two declined to participate as they were not trained to use VRIIIs.

In order to conduct the reflexive meetings, the 10 healthcare practitioners involved at the video-recording stage were approached via email and telephone. Three did not reply, two were not able to participate because of work and family issues, leaving five who agreed to take part.

**Fig. 1:**
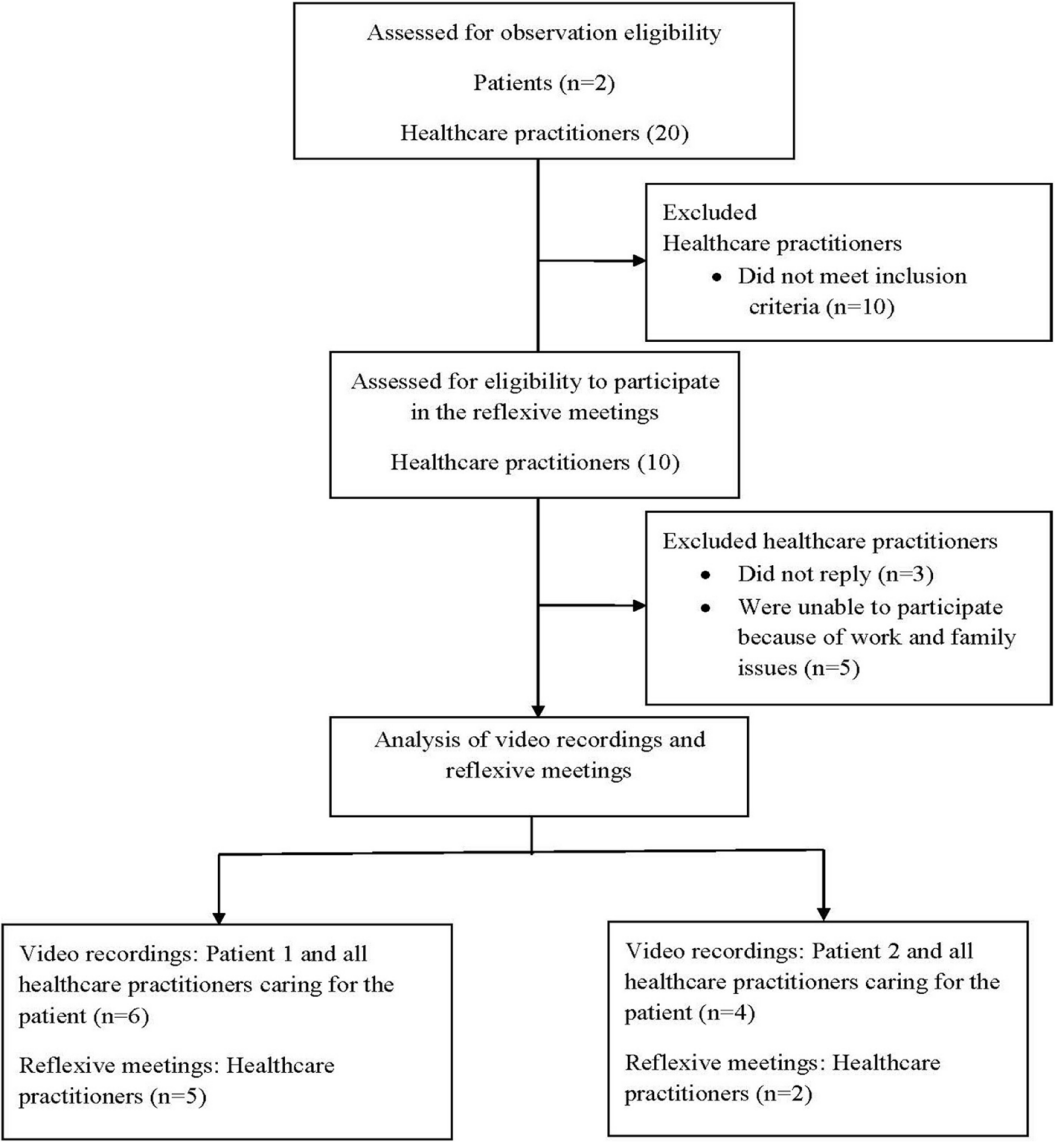
Study flow diagram

Figure 1 summarises the flow diagram of the recruitment and analysis stages of this study.

### Video observations

More than 100 tasks (e.g. administer VRIII and IV fluids) were observed while using VRIII to treat elevated CBG (see Table 3 for examples).

**Table 3:** Summary of the main tasks observed to treat elevated CBG using VRIIIs

The treatment of elevated CBG using VRIIIs started with confirming the potential need for VRIII in the ward round/board meeting. Each morning between 8 am and 10 am there was a ward round during which consultants, registrars, foundation year one doctors, and a senior nurse checked each patient’s status, examined each patient and reviewed their progress, laboratory results, and medications. After the ward round finished at around 10.30 am there was a board meeting attended by various healthcare practitioners including nurses, consultants, registrars, foundation year one doctors, medical students, receptionists, pharmacists, and occupational therapists. The staff discussed patients’ cases and approved their treatment plans. Nurses came to check the confirmed plans for their patients, with a senior nurse documenting the plans on a yellow paper.	
Ensuring the right medications were prescribed, based on CBG readings, was conducted by doctors who were required to prescribe VRIII, IV fluids, and IV glucose, stop all diabetes medicines, except long-acting insulin analogues which were to be continued.	
Before assembling the components of VRIII*,* nurses checked that the medications matched the prescription on the EPR. To assemble the components, several steps were performed including, but not limited to, wiping a blue preparation tray with alcohol wipes, assembling equipment (syringe, extendable administration line, IV administration set, drawing up needles, syringe for the flush, sodium chloride 0.9% ampoule for the flush, chlorhexidine alcohol wipes), and drawing up the solution from the sodium chloride 0.9% ampoule using the blunt fill needle.	
Nurses then recorded on the ePMA the medications to be administered (VRIII and IV fluids). Independent verification of the VRIII/IV fluids was conducted before administering insulin/IV fluids by checking the label on the insulin/IV fluid syringe (name, dose, expiry date), confirming this by signing the details on the EPR.	
Ensuring CBG and blood ketones were within the normal range was conducted by bedside monitoring of CBG and blood ketones every 1–7 h. Nurses were required to regularly monitor cannula, patient complaints, and site of injection, based on the patient’s clinical status.	
Nursing handovers were conducted three times a day for each patient, sometimes during lunch breaks.	
It was clear that electronic documentation of CBG/ketone readings, VRIII rate, insulin/fluids administration, and VIP score was a crucial step by which healthcare practitioners made sure each task they accomplished using the EPR system was documented.	

The researcher noticed that participants were initially trying to modify their work in order to deliver the best care (Hawthorne effect) [23]. However, there was no observable sustained change in participants’ behaviour when they were being filmed. For example, while the nurse initially cleaned her hands per the Aseptic Non-Touch Technique guidelines, after five minutes of recording she no longer used alcohol rub or soap and water but instead applied non-sterile gloves before checking the EPR.

### EPR

Table 4 shows the demographics and the main data concerning CBG monitoring obtained from the two patients’ medical records.

**Table 4:** Demographics and CBG monitoring data from two patients’ medical records

	Patient 1	Patient 2
**Age (years)**	87	84
**Gender**	Male	Male
**Frequency of CBG monitoring (average; range)**	4.5 h; 2–7 h	2 h; 1–3 h
**Percentage of time during which CBG was at target range**	Day 1: 4% of 24 h Day 2: 25% of 24 h	17% of 24 h
**Percentage of CBG readings of < 4.0 mmol/L or > 12 mmol/L**	Day 1: 90% of CBG readings > 12 mmol/L Day 2: 0%	0%
**Duration of persistent hyperglycaemia**	Day 1: persistent hyperglycaemia over 24 h Note: there was no DKA associated with the persistent hyperglycaemia	0
**Incidence of hypoglycaemia episodes**	No episode reported	3 episodes
**Number of times IV glucose infusion was administered for managing hypoglycaemia**	0	2

Most of the time the BG readings of both patients were out of the target range. One patient had persistent hyperglycaemia over 24 h and the other experienced three episodes of hypoglycaemia for which he was treated with two 20% IV glucose infusions.

#### Reflexive meetings

As a result of analysing the reflexive meeting transcripts, three broad themes were identified. (1) Safety strategies: standardise, adapt, and learn to ensure delivery of patient care. (2) Lack of knowledge and insufficient organisational infrastructure as the main challenges in the use of VRIII. (3) Suggestions for enhancing the effectiveness of current safety strategies. (See Supplementary file 2 for the themes, sub-themes, codes, and quotes of the reflexive meetings’ analysis.) These themes are described in turn below.

1. Safety strategies: standardise, adapt, and learn to ensure delivery of patient care

During the reflexive meetings, healthcare practitioners reported that the use of standardised improvements provided by the hospital facilitated the delivery of patient care using VRIIIs. Specifying a time to start the VRIII in the VRIII guidelines, the availability of VRIII guidelines, the use of ready-to-administer insulin infusion syringes, and the availability of the treatment algorithm provided as a laminated printed sheet inside the ‘hypo box’, have together improved the practice of using VRIII. Most participants said that the use of EPR produced more accurate and clearer prescriptions compared to handwritten ones. The importance of using checking and verification was emphasised as a strategy for ensuring patient safety while using VRIII, e.g. independent verification and countersigning before administering the VRIII and fluids, to ensure that nurses get the right information (right dose, expiry rate); checking the CBG before starting the VRIII; and checking the patient’s blood ketones if the CBG level was > 12 mmol/L.

*I think … because insulin is such … can be such a … if you get it wrong it can cause a serious problem, so I still do think that it should be double-checked. Any infusion like that we would get double-checked anyway.* (Nurse 1)

The use of VRE revealed that context-dependent adaptation was the most frequently described and observed strategy used to ensure the delivery of patient care. Most of the time, these context-dependent adaptations had positive outcomes in terms of patient care delivery, leading to the tasks being accomplished. For example, nurses seeking verbal orders for IV glucose when the doctors were busy, and nurses administering IV glucose then checking if it was prescribed to prevent delays in treating hypoglycaemia and consequent patient harm. There were, however, other outcomes that led to harm or inadequate care. One of the inadequate adaptations occurred when, as described by the nurse assistants, if the nurse was busy and the patient had hypoglycaemia, they would give the patient a sugary drink. The nurse assistants were not aware that the patient was on a VRIII and nil by mouth. By being unaware of the VRIII and the patient’s status, giving oral rather than IV glucose might delay the patient going into theatre.

*If it’s not prescribed then I would just speak to the doctor, tell them it’s urgent and if … ask if I can either have a verbal order if they’re not able to prescribe it there and then because it could lead to an emergency if it’s not corrected.* (Nurse 1)

2) Lack of knowledge and insufficient organisational infrastructure as the main challenges in the use of VRIII

The two sub-themes identified were ‘lack of familiarity leads to fear’ and ‘deficient organisational infrastructure risks effective work’.

The sub-theme ‘lack of familiarity leads to fear’ was expressed in the context of prescribing. The most prominent prescribing issues were related to a lack of clinical knowledge about the appropriate IV fluids to use; the need for prescribing long-acting insulin analogues alongside the VRIII; the need to discontinue other diabetes medicines; and the importance of prescribing IV glucose (for hypoglycaemia). Nurses reported that these problems can occur because of a lack of experience (especially seen with new doctors); inadequate training for VRIII indications, side effects, and monitoring; and poor training of senior doctors in how to use electronic systems to prescribe VRIII and IV fluids. This view was echoed by a specialist registrar who described the EPR as a relatively new system and acknowledged that the training to use it was mainly given to junior doctors. Specialist registrars were not used to using it, which could lead to delay or errors in the electronic prescription.

Table 5 shows a vignette that describes the problem with prescribing IV fluids and how it was approached.

**Table 5:**
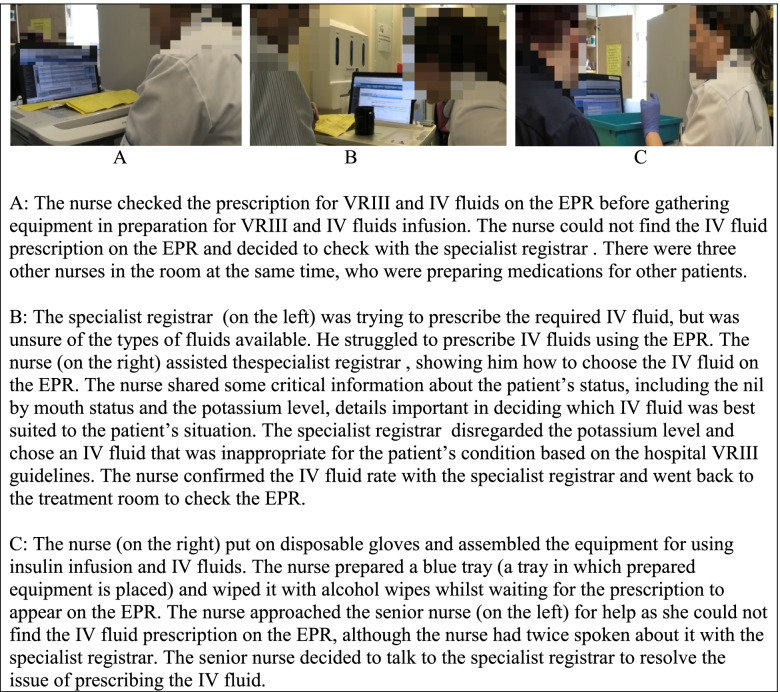
Prescribing IV fluids

Other challenges, such as fear of hypoglycaemia and a lack of confidence treating it, were mainly related to lack of experience, and hesitant new staff. (See Supplementary file 3 for a video clip on confirming the treatment of hypoglycaemia with a senior nurse).

Challenges associated with deficient organisational infrastructure were exemplified by the requirement for frequent CBG monitoring with an insufficient workforce; insufficient knowledge gained from e-learning courses; and the near-patient wireless CBG meter results sometimes not being updated on the EPR.

*Yeah we do struggle sometimes with the equipment because sometimes if after the procedure we put the meter in the [docking] station and it doesn’t really update, I mean the system, it’s [not] updated so sometimes a nurse will ask if the blood sugar testing was done so yes, I tell them that it’s been done but it’s not showing on the computer.* (Nurse Assistant 3)

3) Suggestions for enhancing the effectiveness of current safety strategies

The reflexive meetings allowed healthcare practitioners to suggest solutions that they regarded as being essential to enhancing quality and safety while using VRIIIs. Their suggestions were categorised into staff-focused and system-focused enhancement strategies. Staff-focused strategies included improving staff knowledge about prescribing and monitoring VRIII; predicting the need for a VRIII even if one is not prescribed; using proactive measures to prevent further hypoglycaemia in patients with ongoing risk, e.g. educating healthcare practitioners about the most common symptoms of hypoglycaemia to anticipate the risk; being mindful of the patient’s need for VRIII; and individualising the treatment plan based on the patient’s clinical status, rather than simply following protocols.

*So I guess identifying symptoms of a patient who’s having hypo or hyper will be more helpful in performing this task. What to expect so you know what’s happening.* (Nurse Assistant 3)

System-focused strategies were mainly focused on improving the current training approaches and investing in resources to improve the prescribing of VRIIIs. A common suggestion for improving training sessions was directed towards conducting more specific training on VRIII prescribing, recognising the side effects of VRIII, and how to deal with its side effects. When asked about training to enhance knowledge, the participants were unanimous in the view that face-to-face or in-house training would be much better than e-learning.

*If we have physical training, like... physical is better than just e-learning with just reading, reading, and answering the questions.* (Nurse Assistant 1)

The reflexive meetings ensured the engagement of the participants in the process of elaboration and collective understanding of their perspectives and experiences. Rather than only reporting their experiences descriptively through interviews, the use of video clips during the reflexive meetings enabled the participants to share the meanings of the reality surrounding them and the events of their own lives, without these being interpreted arbitrarily only by the interviewer. This was demonstrated by the example of one participant who realised, while watching the video clips, that he was unaware he had a problem using the EPR to prescribe fluids. Having watched and discussed the video clip, he proceeded to question his own competency using EPR to prescribe, realising that he needed more training in doing so.

## Discussion

This study was the first to explore the use of VRIII in a Vascular Surgery Unit using VRE. The results of this study demonstrated that VRE methodology uncovered the actual and potential hidden complexity in everyday work that encompasses various types of tasks and clarified how it was handled in situ. Engendering healthcare practitioners’ reflexivity helped the researcher to explore how and why some tasks were accomplished in particular ways and to align more closely with the reality of everyday work, the result being a better understanding of WAD while using VRIII. Various methods had previously been used to explore WAD and resilience in healthcare, e.g. field observation, interview, and focus groups [24–26]. These methods can partially capture an approach to understanding WAD. However, no studies had been conducted to explore WAD in the use of VRIIIs using a VRE methodology in which healthcare practitioners are involved in reviewing, analysing, and reflecting on their work.

### Exploring the use of VRIIIs using VRE

Although the study findings were obtained on the basis of video-recordings of two patients and 10 healthcare practitioners, the use of VRE provided data that enabled analysis of VRIII use, and in turn recommendations to improve its use. A range of strategies to ensure the delivery of patient care was observed being used in the system. These included strategies based on the Safety-I approach (e.g. relying on guidelines) for VRIII, as well as strategies derived from the Safety-II way of thinking (e.g. context-dependent adaptations such as delegating the CBG monitoring task to colleagues when the nurse was busy). Practitioners reported that strategies from the Safety-I approach (e.g. independent verifications) were crucial while using VRIII. They also appreciated the importance of context-dependent adaptations (Safety-II) as a strategy for dealing with unexpected situations to ensure patient safety. This finding broadly supports the work of other studies demonstrating how adaptations are required in everyday work to provide safety improvements and resilience in the system [27–29]. It is imperative to highlight that the RHC concept has often been misconstrued, as adaptations have been regarded as being opposite to control, the implication being that the two cannot coexist [30, 31]. This study found that participants used a comprehensive approach based on two principal strategies (Safety-I and Safety-II) to strengthen systems and enhance their resilience and safety.

Previous studies highlighted the impact of using VRE in various clinical settings by demonstrating achievements such as enabling participants to be explicit about their own practices and problems in healthcare-associated infections [32], developing meaningful solutions for problems in an intensive care unit [33], and enhancing team capacity to enact person-centred care to improve dementia care [34]. In this study, VRE helped healthcare practitioners to be reflexive and explorative of challenges, acknowledging that the work may need changing and suggesting practical solutions tailored to their work.

The use of VRE highlighted some of the challenges experienced by healthcare practitioners when using VRIII. These related to a lack of clinical knowledge and experience when prescribing the appropriate IV fluids, failure to appreciate the necessity of continuing to administer long-acting insulin, or lack of awareness of the type of fluids available. Rickard et al. identified that 64% of the IV fluids prescribed with VRIII did not have the recommended potassium concentration [35]. This is consistent with what was observed in this study where there was a discrepancy between the fluid prescription and patients’ clinical status. Another challenge identified in this study was the use of the ePMA to prescribe VRIII and IV fluids. This finding is likely to be related to the fact that, prescribers rotate from other organisations, so the ePMA system might be new to them. This explanation matches the conclusion reached in other NHS Trusts, where the initial implementation of the ePMA system was more time consuming than the paper method, although as staff became more familiar with it, the process of prescribing, monitoring, and administering became more efficient [36].

The study hospital used different ways to improve staff knowledge and enhance patient safety, however, some participants said there was no specific training on VRIII use or its complications. The healthcare practitioners stated that completing the diabetes e-learning module with a pass did not necessarily mean they had gained the practical benefits expected with completing the module. With this in mind, some suggested that face-to-face in-house diabetes and VRIII-focused training sessions, tailored to the practical aspects of their work, would be more effective than e-learning.

Monitoring CBG every hour proved hard to achieve given the increased workload and shortage of nurses. One suggestion could be around providing clearer guidance on an acceptable window for BG monitoring frequency based on the patient’s case and the stability of their BG readings. For example, monitor BG every hour; if four consecutive readings are within the target range, then reduce the frequency of monitoring to two-hourly and return to hourly monitoring if BG moves outside the target range [37]. Therefore, it is crucial that healthcare practitioners themselves define the scope of their work with the clarity needed for harmonised, tailored training and development plans [38]. Understanding WAD and the needs of healthcare practitioners may ease the demand on system resources as well as satisfying healthcare practitioners’ needs.

Given the complexity that was found in the use of VRIII and how unpredictable situations emerged, it is vital to think about using methods focusing on engaging healthcare practitioners to explore the taken-for-granted work, e.g. VRE [34], in order to understand and investigate complexity in everyday work. Translating these methods into healthcare is difficult to achieve with the resources currently available. The NHS needs a clear shift in its safety strategy towards embedding safety professionals who are aware of the depth and breadth of these methods and are able to act as part of a healthcare system in which they are able to learn from adaptations and adjustments, suggest more context-related solutions and interventions, and engage in system design which will in turn help in realigning WAI and WAD and decreasing the gap between the two [39, 40].

### Strengths and limitations of the study

Quality and safety improvement initiatives cannot be understood outside their context, and initiatives can only influence work when healthcare practitioners agree that strategies proposed for implementing these initiatives will improve their work [41]. The novelty of the present study’s use of VRE methodology lies in the fact that its innovations arise from within established work (exnovation), and from within practitioners’ collective sense-making of their work.

This study identified several strategies that might enhance safety in the use of VRIII in the study hospital, including improving electronic prescriptions by providing preparatory training sessions for senior and junior doctors; face-to-face training; and teaching sessions on VRIIIs focused on the practical needs of healthcare practitioners. CBG monitoring and independent verification of prescribing and administering VRIIIs are important strategies for enhancing safety, meaning that attention should be directed towards investing resources in ensuring the consistency and continuity of experienced staff over time. The study’s findings could potentially be discussed with healthcare practitioners, NHS safety professionals and diabetes guideline developers, with a view to exploring the applicability of the suggested solutions and assessing how they might influence future VRIII guidelines and policies.

Patient and public involvement is recommended as best practice. Although there was no patient involvement in this study, healthcare practitioners were proactively involved in the study conduct and analysis, engaged in designing ways to video themselves and patients as well as analysed their work in the reflexive meetings, a process revealing opportunities for improvement by learning lessons from the everyday work that would otherwise remain undetected.

The small number of patients engaged was one of this study’s limitations. Conducting applied research in a busy tertiary teaching hospital was challenging and recruitment relied on the occurrences of acute patient cases, their prompt identification and notification by the study collaborator and the pharmacy team to the researcher.

Reflexive meetings in reported VRE studies were usually attended by a group of participants who had been involved in the video clip [42–44]. A hallmark of an effective study is that its method can be adapted as and when necessary to achieve the most promising results [45, 46]. In this study, adaptation needed to be made in relation to conducting the video reflexive meetings because of the COVID-19 pandemic. Although there were initial concerns that having only one healthcare practitioner in each reflexive meeting might limit the depth of the discussion, it was found that healthcare practitioners were very keen to discuss the video clips, openly analyse their work and suggest solutions for improving the delivery of patient care.

Although the number of participants (patients and healthcare practitioners) was considered low, the use of a mixed method approach to explore a specific phenomenon, i.e. the use of VRE in a Vascular Surgery Unit, along with engaging participants in analysis of their own work, enhanced the credibility and transferability of the study findings.

A key concern with using video approaches is the effect they may have on practice and on the communication between the participants and the patients on one hand, and between the participants and their colleagues on the other. Existing literature confirmed that there is no evidence video-recording causes significant alteration to the way participants usually behave [23, 47]. Although at the beginning of the study some healthcare practitioners made some adjustments to their work because of the mere presence of the researcher’s handheld camera, the prolonged presence of the researcher in the field unit helped the healthcare practitioners get accustomed to the camera. Using a chest-mounted camera would likely have made the recording more efficient by enabling hands-free recording and filming from a different angle, without making healthcare practitioners conscious that a researcher was observing and filming their work.

We are aware that the current findings of this study provided snapshots of how VRIII was used to treat elevated CBG. Future work should focus on collecting more data to develop a richer and deeper view of the use of VRIII. Additional studies using VRE need to tap into a wider range of patients and healthcare practitioners across many units in the same hospital used for this study and across different Trusts, to build a more comprehensive understanding of WAD in the use of VRIII and thus better inform policy making and clinical work.

## Conclusions

To the best of our knowledge, this study is the first to have explored the use VRE to understand how everyday work is done in reference to the use of VRIII. VRE had the utility to explore everyday work and to add depth to understanding how everyday tasks were accomplished. It also generated increased reflexivity in healthcare practitioners, which was a precursor to enhancing their knowledge and insights into how to work in complex systems by proposing practical solutions tailored to their needs.

## Supplementary Information

**Additional file 1.**

**Additional file 2.**

**Additional file 3.**

## Data Availability

The unpublished data used in the current study are available from the corresponding author on request.

## Abbreviations

BG: Blood glucose
COVID-19: Coronavirus disease
EPR: Electronic Patient Record
IV: Intravenous
NaDIA: The National Diabetes Inpatient Audit
NHS: The National Health Service
RHC: Resilient Health Care
VRE: Video Reflexive Ethnography
VRIII: Variable rate intravenous insulin infusion
WAD: Work as Done
WAI: Work as Imagined
