# Supplementary material for: Using video reflexive ethnography to explore the use of variable rate intravenous insulin infusions
Source: BMC Health Serv Res. 2022 Apr 23;22:545. doi: 10.1186/s12913-022-07883-w (PMC9034771; doi:10.1186/s12913-022-07883-w)
Supplement: Supplementary file 1 — Additional file 1. [file 12913_2022_7883_MOESM1_ESM.doc]

**S1 File. Reflexive meeting discussion guide.**

1. Welcome

- I would like to acknowledge and thank you for agreeing to participate in this part of the study and to discuss your daily practice for the benefit of your patients and hospital. You know that I have videoed your practices while using variable rate intravenous insulin infusion (VRIII) and today I would like to show you some video clips, listen to you, know your thoughts about the ordinary work and identify a realistic solutions and recommendations to improve your work and patient safety in the use of VRIII.

1. Ground Rules

- To maintain confidentiality I would ask that information provided in the reflexive meeting discussion to be kept confidential. Please do not discuss or share with others beyond this group, what is said in this meeting.
- The research team based at University of Reading will handle confidentiality of all identifiable information (names, email and/or telephone numbers, voices).
- I anticipate this meeting to last for approximately 60 minutes.
- Are you still happy to do this and audio-recording the meeting?

1. **Turn on audio-recorder**
2. Let’s begin:

- Tell me about what we’ve just seen in this clip.
- Is this something occurs often, if this happen, what do you do?
- What you are trying to do (goal)?
- Tell me more about this activity (e.g. independent verification).
- Tell me who and/or what was involved – and why were they involved?
- How do you normally do it in practice?
- Are there times, has it gone well when you did this task and tell me about what happened.
- Is there anything that can make this better? If we were to enrich this further, what actions need to happen now?

We talked about various clips, and I know that your work is more than these. Focusing on using VRIII to treat elevated blood glucose, can you talk about key points that you would like to highlight.

I want to thank you for taking time out of your busy schedules to discuss the clips with me today.
