# Supplementary material for: Using video reflexive ethnography to explore the use of variable rate intravenous insulin infusions
Source: BMC Health Serv Res. 2022 Apr 23;22:545. doi: 10.1186/s12913-022-07883-w (PMC9034771; doi:10.1186/s12913-022-07883-w)
Supplement: Supplementary file 2 — Additional file 2. [file 12913_2022_7883_MOESM2_ESM.docx]

**S2 Table. Themes, sub-themes, codes and illustrative quotes for the reflexive meetings' analysis.**

| **Themes** | **Sub-themes** | **Codes and illustrative quotes** |
| --- | --- | --- |
| **Lack of knowledge and insufficient organisational infrastructure as the main challenges in the use of VRIII** | **Lack of familiarity leads to fear** | - Lack of knowledge of the appropriate IV fluid or its availability.   *'I think the doctors need to be a bit more, not proactive, but a bit more knowledgeable about prescribing them... If they have a bit more training or knowledge about how to prescribe it correctly and making sure, as well, that there’s some IV glucose prescribed in case.' (N1)*   - Seniors do not use EPR as frequently as juniors.   *'And because I’m always been used to just do a handwritten prescription, and this system is relatively new, so that’s why we’re just looking for the system. And I don’t use it as frequently as other junior doctors, so mainly it’s done by junior doctors because they use it more frequently and they’re more slick at finding it'. (D1)*   - Fear of hypoglycaemia.   *'She would… yeah, she didn’t really need to get my advice, but, I mean, you would… the nurse themselves would be responsible for getting the glucose and starting it on the patient and checking it’s prescribed…'. (N1)* |
|  | **Organisational infrastructure risks effective work** | - The policy of frequent monitoring is challenging.   *'The main thing of using them is having to check the blood sugar every hour is… it can be quite… well, it’s difficult to be able to go back the patient at the same time every hour, that’s why… well, we do delegate it to the carers, the health care assistants'. (N1)*   - The need for more time to use the EPR.   *'For me, if I want to use it, I might take a little bit more time just to find where the… because I don’t use it as frequently. It’s just finding… knowing how to use that system and getting enough training… proper training on the use of the electronic system itself.' (D1)*   - Not enough understanding with e-learning.   *'Because the one we do is just with the computer [e-learning]. So, if we have like a... if we have physical training, like... physical is better than just e-learning with the... just with reading, reading and answering the questions.' (NA1)*   - Struggle with technical problems while using the bedside (point of care) testing meters.   *'Yeah we do struggle sometimes with the equipment because sometimes if after the procedure we put the metre in the [docking] station and it doesn’t really update,I mean the system, it’s [not] updated so sometimes a nurse will ask if the blood sugar testing was done so yes, I tell them that it’s been done but it’s not showing on the computer.' (NA3)*   - Covid-19 reduces the number of training sessions/opportunities?.   *'But, yeah, we have been having… before the whole Covid situation, we were having quite a lot of teaching sessions from the diabetic specialist nurses.' (N1)*   - Low workforce increases workload   *'Like, we are so… in the ward, we are so, so busy, like, very busy, sometimes it’s nearly 12 o’clock and we didn’t do a wash; we didn’t do any blood sugar.' (NA2)* |
| **Safety strategies: standardise, adapt, and learn to ensure delivery of patient care** | **Context-dependent adaptations to deliver patient care** | - Verbal orders for urgent cases   *'If it’s not prescribed then I would just speak to the doctor, tell them it’s urgent and if… ask if I can either have a verbal order if they’re not able to prescribe it there and then because it could lead to an emergency if it’s not corrected.' (N1)*   - Any nurse available to countersign on the system.   *'I think it looks like my colleague’s waiting for me to come and countersign on the computer that they were able to put the variable rate up, but I was busy doing something else. I’m not sure… I don’t know if it show… they got somebody else to do it, or it might have been me, I’m not sure.' (N1)*   - Administer IV glucose then check if it is prescribed.   *‘If it was me, then I probably would have gone straight to the hypo box, put the glucose up, then checked that it was prescribed and make sure the doctor prescribed it, but I know other nurses would… and I wanted to just… with my colleague I advised her to check it was prescribed first, just because it’s her PIN number, not mine.' (N1)*   - If nurses are busy, NA gives oral glucose   *'Yeah, I just straight away let them know and, if they’re busy, if the blood sugar’s very, very low, we have to give out glucose.' (NA2)*   - Keeping hand-written results as backups.   *'I tell them that it’s been done but it’s not showing on the computer. So I always make sure that I have released all the results so that they can just check the paper and then they will put it manually on line.' (NA3)*   - Acquire BG testing strips from other ward if there is shortage.   *'Yeah, although sometimes we don’t have enough strips so we go to the other ward to get some.' (NA3)* |
|  | **Standardised practice leads to easier, consistent and safer use of VRIII** | - Quick preparation because of the use of prefilled insulin syringes.   *'So because the syringes come premade, we just have to make sure that we’ve got the right… well, it already comes premade in the correct dosage and concentration, but we just have to just double check that it’s correct. ' (N1)*   - Guidelines now specify when to start VRIII.   *'Usually for patients who for example are fasted for theatre… diabetic patients are fasted for theatre… so… so there is a protocol that is there in the… and used in the hospital.' (D1)*   - Algorithm on the hypo box to treat hypoglycaemia.   *'We have like the algorithm in there [hypo box], so you can… you could have gone straight to get that and do, you know, follow the protocol. Excuse me, follow the protocol through that.' (N1)*   - EPR as a way to ease prescriping VRIII and IV fluid.   *'It’s all done on the computer. So you have to open the EPR, which is the Electronic Patient Record, and you find links on them that tells you where… how to prescribe and then you do the actual prescription.' (D1)*   - Automatic documentation of BG results.   *'Also, now it’s on the computer the… how you document the blood sugars. The blood sugars come automatically on the system.' (D1)*   - More accurate prescription with EPR compared to hand written prescription.   *'So… the prescription… this electronic system ensures more that… yeah, that the prescription is done more accurately and hopefully no mistakes are done or gives a clearer… clearer to read, clearer to document.' (D1)*   - Policy of medication information checking   *'Also, checking the expiry date to make sure that it’s not… obviously, not expired, and then we check… I wrote the date and the time that we started the… well, that we opened the syringe on it and, yeah, just, yeah, just double checks really.' (N1)*   - Policy of double independent verification before administration   *'Well, we… it… normally in practise we get two people to set it up and then one person takes it to the patient and then, after it’s all connected, the… both of us sign on the computer.' (N1)*   - Monitor BG, and ketones   *'For patients who are nil by mouth usually they check it every hour. ' (NA3)*  *'Then we took some... some blood, then we check the... the glucose, if it’s... if it’s higher than 12, so automatically we check ketones.' (NA1)*   - Patient identification   *'So when I approached a patient I check with the patient, I mean the identity of the patient.' (NA3)* |
|  | **Training and teamwork to improve knowledge and patient care** | - Training by shadowing and observing senior colleagues.   *'So, yeah, the... the nurse, she taught... she taught him how to do it and he practice with me. But the first... how and what, is always with the practice... practitioner nurse and, like, shadowing me, like, one or two days. And after that, he can be allowed to do it with present of... you know, as with if he’s new starting.' (NA1)*   - Considerable a mount of teaching sessions with diabetic nurses.   *'Well, education really. We do have… we have a lot of teaching sessions with the diabetic nurses.' (NA1)*   - Posters of all type of insulin.   *'We’ve got a poster at the… in the treatment room as well of the types of insulin and you must continue long acting alongside the variable rate.' (N1)*   - Electronic learning.   *'Because the one we do, it just asks with the computer. So, we need to read... and we need to read and answer, so maybe we can like... maybe we can read and we can answer, good answer, but we don’t... we don’t understanding what that really means.' (NA1)*   - Experience and vigilance to predict hypoglycaemia.   *'Because one time I think it was early in the morning and I went to check a patient who I know was diabetic and he was feeling… looking a bit strange, so I just checked the blood sugar right away because I was thinking that he might have hypo, at that time, so I just checked it myself. And then he’s having hypo, so I told any one of the nurses right away that he…' (NA3)*   - Teamwork to care for patients on VRIII   *'Of course, everybody who’s caring for the patient on the ward, and that includes the doctors caring for the patients. Usually there is a team of doctors. And the nurses as well'. (D1)*  *'So all I can do is just give assistance to the nurse looking after the patient, just to keep an eye on other symptoms as well.' (NA3)* |
| **Suggestions for enhancing the effectiveness of current safety strategies** | **Focused and face to face training for better use of VRIII** | - More focused training on how to prescribe VRIII, fluids and IV glucose.   *'If they have a bit more training or knowledge about how to prescribe… prescribe it correctly and making sure, as well, that there’s some IV glucose prescribed in case.' (N1)*   - More training on VRIII indications.   *'I think not… not much [far] from clear prescription and… and careful monitoring, and also [maybe] good indication, because not all these patients are fasting for theatre… diabetic fasting for theatre, need that insulin infusion.' (D1)*   - More training on hypo and hyper symptoms.   *'So I guess identifying symptoms of a patient who’s having hypo or hyper will be more helpful in performing this task. What to expect so you know what’s happening.' (NA3)*   - Face to face training.   *'So, if we have like a... if we have physical training, like... physical is better than just e-learning with the... just with reading, reading and answering the questions.' (NA1)*   - Further training specific for diabetes.   *'Well, I guess we… we should be given further trainings as well about diabetes so that we are more helpful in the treatment of patients with diabetes.' (NA3)* |
|  | **Experience and effective communication makes the use of VRIII easier** | - More experience is making things easier.   *'I’m experienced now in doing them, you know, in preparing them because I’ve been doing it for years, and it’s… I know where to get all the things, I know how to do it and, you know, I know how to manage it. So I think the more you do it the more you get used to it, really.' (N1)*   - Being mindful to predict when to start VRIII   *'So we just have to be really mindful that, actually, the patient is diabetic, they’re on insulin, they should… if they miss two meals then we know, yes, they should really start on the sliding scale variable rate.' (N1)*   - Thinking about patient case rather than just follow protocols.   *'It depends on their sugar level and how long they are going too fast. So I always think about these things rather than just following protocol.' (D1)*   - Better communication   *'I think it’s just working in… better communication between the nurses and the doctors about this sort of thing.' (N1)* |
|  | **Standardisation and automation to ensure patient safety** | - Importance of double checking, high risk medication   *'So, I think even… even only… well, having one nurse, I don’t think it would be very safe. Just because you do need someone to check that it’s all set up correctly.' (N1)*   - Add a reminder to the EPR-note to tell what to/not to give with VRIII.   *'So I think it’s just continue that and to sort of remind the nurses really, or, maybe, on the chart, or, you know, when they… when the doctor’s prescribing the certain insulin it should have a little note saying must be given or do not give alongside variable rate, or something like that.' (N1)* |

*D* Doctor, *N* Nurse, *NA* Nurse assistant
